# PREPARING STUDENTS FOR A MULTIGENERATIONAL WORKFORCE: PERSPECTIVES FROM OLDER WORKERS AND RETIREES

**DOI:** 10.1093/geroni/igad104.0471

**Published:** 2023-12-21

**Authors:** Allyson Graf, Katherina Nikzad-Terhune

**Affiliations:** Northern Kentucky University, Highland Heights, Kentucky, United States; Northern Kentucky University, Highland Heights, Kentucky, United States

## Abstract

Workforce diversity can be a major asset for personal, interpersonal, and economic development. Age diversity is prevalent with five generations currently represented in the workforce. Intergenerational tension is common, however, largely fueled by stereotypes centered on age and generation that can infiltrate all workplace processes. Working from the Age-Friendly University framework, our aim was to gather qualitative data to inform the development of a training program for college students, pairing information on age-related bias with opportunities for meaningful intergenerational exchange to mimic workplace interactions. We hosted three community-based focus groups with adults ranging from 60-69 years of age who were either currently employed or recently retired (N = 10) to discuss their intergenerational interactions in the workplace. Participants had an average of 41.06 years of work experience and worked across multiple industries, most commonly administrative work, food services, government, and healthcare. The most common theme reported was the dismissal of participants’ work experience by their younger colleagues, which some equated to a lack of respect due to their age, and others painted as a breakdown in communication. Technology was highlighted as exasperating intergenerational tensions. Participants also reported that younger colleagues often approached them for general life advice, which helped to reduce tensions. The willingness to see the value of older adults’ wisdom in general, but not specific to the work at hand, has implications for the content and structure of future career-readiness training programs. Recommendations for enhancing positive intergenerational exchanges and reducing generational tensions within the workplace will be discussed.

